# Network Latency in Teleoperation of Connected and Autonomous Vehicles: A Review of Trends, Challenges, and Mitigation Strategies

**DOI:** 10.3390/s24123957

**Published:** 2024-06-18

**Authors:** Sidharth Bhanu Kamtam, Qian Lu, Faouzi Bouali, Olivier C. L. Haas, Stewart Birrell

**Affiliations:** Centre for Future Transport and Cities (CFTC), Coventry University, Coventry CV1 5FB, UK; ad6501@coventry.ac.uk (F.B.); o.haas@coventry.ac.uk (O.C.L.H.); ad2998@coventry.ac.uk (S.B.)

**Keywords:** network latency, teleoperation, connected and autonomous vehicle (CAV), latency mitigation strategies—perception and control

## Abstract

With remarkable advancements in the development of connected and autonomous vehicles (CAVs), the integration of teleoperation has become crucial for improving safety and operational efficiency. However, teleoperation faces substantial challenges, with network latency being a critical factor influencing its performance. This survey paper explores the impact of network latency along with state-of-the-art mitigation/compensation approaches. It examines cascading effects on teleoperation communication links (i.e., uplink and downlink) and how delays in data transmission affect the real-time perception and decision-making of operators. By elucidating the challenges and available mitigation strategies, the paper offers valuable insights for researchers, engineers, and practitioners working towards the seamless integration of teleoperation in the evolving landscape of CAVs.

## 1. Introduction

CAVs are continuously developing to provide safer and more convenient transportation. These vehicles can prevent many road accidents due to human errors [1]. The Society of Automotive Engineers (SAE) J3016 (2016) defines six levels of driving autonomy for on-road CAVs, from level 0 to level 5 [1]. Higher levels have more automated driving features, making them entirely driverless vehicles [1,2]. In levels 0 to 2, a human driver is required to be present at all times. These levels have supporting/assisting features (e.g., warning systems). In level 3, human drivers are also present. Still, these vehicles are said to be conditionally automated, i.e., the vehicle can handle a few situations (such as lane changing, autonomous emergency braking (AEB)), and the driver should be monitoring and ready to take over the vehicle at any instant. Level 4, which includes higher automation features, has an automated driving system (ADS) that can handle most dynamic driving tasks without human intervention [1,3]. Ultimately, the vehicles are expected to handle all situations and become fully autonomous in SAE level 5. Today, level 2–4 vehicles are being tested and deployed in the market [3,4]. For example, Tesla’s level 2 with autopilot system [5] and level 3 with the fully self-driving mode [6] are commercially available [1,3].

Even with remarkable advancements in CAV development, it would be unrealistic to anticipate zero system failures. It is widely acknowledged that CAVs may not handle all road incidents and will depend on human decisions [7], as the real world can be very uncertain. In this case, a human operator is required to take over the vehicle. It may not be through physical access but by remotely assisting/operating the vehicles in challenging situations [1]. This remote access is known as teleoperation. The word ‘tele’ comes from a Greek word meaning “at a distance” [8]. It involves a remote human operator to monitor, assist, and control a vehicle from a distant location for some manoeuvres or edge situations [7]. For CAVs, it is also known as remote driving or tele-driving.

One particular use case of remote driving to enhance vehicle safety is by bringing the vehicle to the minimal risk condition (MRC), a stable and safe stopped state when a problem occurs [1]. Additionally, integrating human teleoperators in the over-the-network control loop can improve the vehicle’s operational efficiency by broadening the vehicle’s operational design domain (ODD) allowing it to cope with challenging environmental conditions such as weather, visibility, speed limits, types of roadway, and motorways [3].

The communication system is an essential aspect of teleoperation. Maintaining the safety and security of the teleoperation communication systems is important, as these systems should be uninterrupted and transmit data in real time [9]. The British Standards Institution (BSI) Flex 1886 recommends that ADS have appropriate autonomous capabilities to perform a minimal risk manoeuvre (MRM) safely (as a fallback solution to achieve an MRC) when connectivity is compromised or lost. The communication system can be compromised by system malfunction/failure, cyber-attack, or simply network issues (e.g., latency or bandwidth constraints). The communication capabilities of fourth-generation (4G), fifth-generation (5G), and future generations of wireless networks can facilitate the remote driving of CAVs [3]. A few companies have already demonstrated that 5G networks are reliable for the teleoperation of remote vehicles, and they expect promising results in future experiments [10]. Various networking technologies, including Ethernet, wireless fidelity (WiFi), General Packet Radio Service (GPRS), third generation (3G), long-term evolution (LTE), 4G, and 5G [10], have been considered in the literature through real-world implementations, simulations, and emulations. However, ensuring the availability of 5G and newer technologies on every road at all times might not be realistically achievable [10]. Similarly, the availability of remote operators 24/7 might be an issue. Moreover, the CAVs are expected to generate approximately 10 gigabytes (GB) of data every minute [11], suggesting that even advanced networks such as 5G will require optimisation [10].

Network latency or delay is one of the major challenges that can cause disruptions in communication systems [1,3,7,8,9,10,12]. Latency can affect the quality of sensory data from the vehicle to the remote station and control command data from the station to the vehicle, in turn degrading the operator’s and teleoperation performance. It can also cause over- and under-steering of the remote vehicle. Moreover, longer and variable (time-varying) latency is an even more significant problem, making the control problem very challenging. The remote operators can tolerate latency up to a threshold beyond which it becomes unmanageable. For example, during vehicle teleoperation, Zhang [3] states that a constant latency of less than 170 milliseconds (ms) has a minor impact and is easily manageable whilst the operator can adapt to latency below 300 ms. However, latencies (with less variance) between 300 ms to 500 ms become challenging and significant for the operator to handle the vehicle (at slow speeds). Latencies above 700 ms make it nearly impossible for the teleoperator to interact with the vehicle in a timely manner.

### 1.1. Comparison with Existing Surveys

A summary comparison with relevant survey papers is provided in Table 1. Several related survey papers have contributed to the realm of teleoperation, focusing on aspects such as teleoperation research scope, architectures, taxonomies, advanced communication links, and challenges of mobile robots [8,12,13] and CAVs [1,3,7,10,14,15].

Opiyo et al. [13] emphasise architecture, communication links, and situational awareness in teleoperation. While some studies present teleoperation methods and enhancement techniques, Farajiparvar et al. [12] review approaches for time delay mitigation, focusing on control theory, user interface designs, and time-series prediction models. Similarly, Moniruzzaman et al. [8] comprehensively review teleoperation challenges and examine existing teleoperation methods and enhancement techniques (with limited coverage of latency compensation techniques). However, these survey papers revolve around mobile robots, which may not be valid for CAVs.

Other recent survey studies focus on CAV teleoperation. For instance, Bogdoll et al. [14] provide a taxonomy for the teleoperation systems, outlining recent terminology in the field. Likewise, Mutzenich et al. [1] stress integrating remote operator roles into industry-standard taxonomies and use cases for regulatory frameworks. They also highlight the importance of designing control interfaces to maximise operator situational awareness. Additionally, Zhang [3] presents a vision of intelligent teleoperation systems powered by artificial intelligence and advanced networks (e.g., 5G), discussing their potential benefits and challenges. Tener and Lanir [7] analyse significant challenges in CAV teleoperation and provide design guidelines for future teleoperation interfaces. Amador et al. [10] compile works on the remote operation of road vehicles, categorising them based on the level of human intervention and identifying challenges in deployment across technological, regulatory, and commercial domains. Zhao et al. [15] survey remote driving challenges and solutions of latency, driving feedback, support control, and initiatives. They briefly provide the impacts of latency on remote drivers and review a few mitigation strategies. Although the existing surveys acknowledge the significance and challenges in the context of CAV teleoperation, extensive research focusing on network latency as a challenge and mitigation strategies specific to this domain remains scarce. Many of these surveys have insufficiently covered the network latency aspects of CAV teleoperation.

### 1.2. Contributions

To the best of our knowledge, the current literature lacks comprehensive studies on the impact of network latency and mitigation methods during teleoperation, particularly in the CAV domain. this paper proposes a comprehensive and systematic review that explores the trends, impact, and state-of-the-art latency mitigation strategies that apply to CAV teleoperation, further offering valuable insights for researchers and practitioners in this field. The contributions of this paper are as follows:

- It thoroughly examines the various network quality-of-service (QoS) parameters and their relation with latency, outlining their significance in the context of teleoperation, further indicating the sources of latency in system aspects of CAV teleoperation.
- It reviews various approaches to model network delays for teleoperation experiments.
- It identifies and delves into various wireless technologies relevant to teleoperation, further analysing their suitability and implications for CAVs.
- It provides insights into various teleoperation practices across different domains, highlighting the unique requirements of remote driving.
- It critically analyses state-of-the-art latency mitigation methods for control and perception latency, discussing their advantages and disadvantages for compensating time-varying delays during CAV teleoperation. Additionally, it presents up-to-date initiatives and standardisation efforts that have contributed to remote driving.

### 1.3. Paper Structure

**Figure 1:**
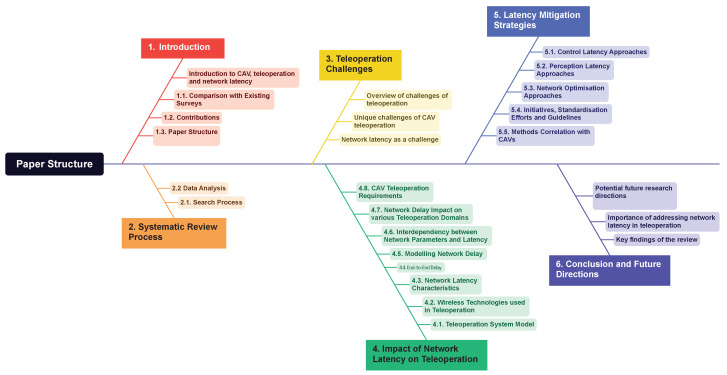
Summary diagram showing the structure of this paper.

The organisation and structure of the paper is shown in Figure 1. Section 2 covers the systematic review process. Section 3 discusses the challenges of teleoperation with a focus on network latency. Section 4 provides the nature of network latency and its impact on various teleoperation domains and finally on CAVs. Section 5 discusses the trends and limitations of strategies for mitigating latency, case studies, and industry initiatives. Finally, Section 6 discusses the key findings of this survey, potential emerging technologies, and methods that can be used as mitigation strategies for CAVs.

## 2. Systematic Review Process

### 2.1. Search Process

**Figure 2:**
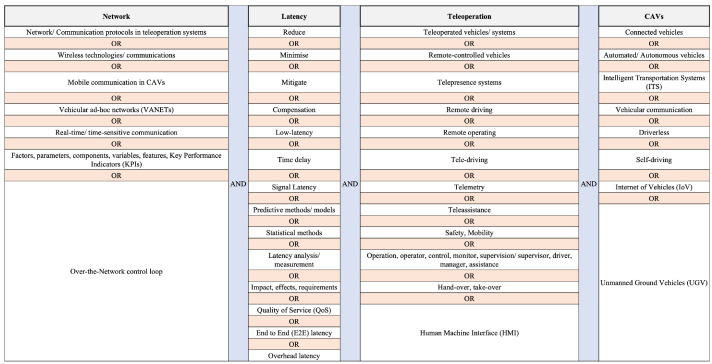
Keywords for search criteria.

The literature for this survey was selected based on various inclusion and exclusion criteria. The papers were searched and classified mainly on relevance to the topic, focusing on keywords such as network latency, teleoperation, and CAVs. The search process included alternate keywords (in different combinations) as shown in Figure 2. However, in this process, it became apparent that a limited number of papers focused on addressing the requirements of the teleoperation of CAVs. To cover this literature gap, papers on teleoperation from diverse domains have been considered and compared with the CAV requirements.

**Figure 3:**
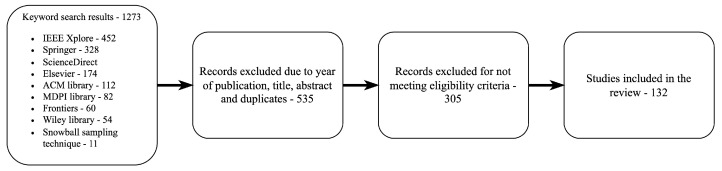
Systematic review selection process flowchart.

The selection process for this review was performed according to the PRISMA guidelines [16] and is depicted in Figure 3. Most of the screened papers are from secondary sources that are peer-reviewed and published articles, surveys, journals, and conference papers, including a few from the non-academic literature from the policy landscape, such as technical reports and white papers. The papers include studies with diverse research methodologies such as frameworks, simulation-based, theoretical analysis, experimental and empirical studies to provide a comprehensive overview. Another criterion was to include literature published within a specified time frame to maintain timeliness. This survey covers literature within the last ten years.

The literature was gathered and acquired from publicly available research databases and search engines such as Google Scholar, BASE, CORE, Science Gov, refseek, ACM library, ScienceDirect Elsevier, Springer, Semantic Scholar, MDPI library, ResearchGate, IEEE Xplore digital library, Wiley library, and Frontiers.

### 2.2. Data Analysis

The total number of papers collected was approximately 230. Among these, about 25% were excluded/screened out, considering their relevance to the inclusion and exclusion criteria. The remaining papers were reviewed and included in this survey paper.

**Figure 4:**
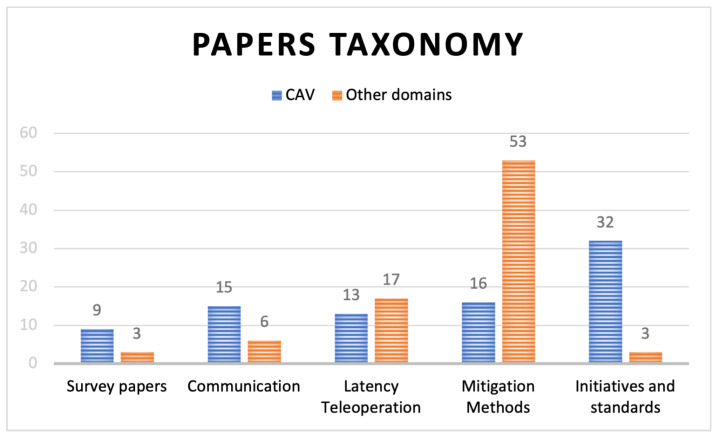
Paper taxonomy classified for CAVs and other domains.

Using different combinations of the keywords mentioned above, the included papers were further categorised into broad topics, including survey papers in relevant fields, papers from networking and communications, papers that focus on network latency and teleoperation, papers on latency mitigation methods, and industry initiatives and standard reports, as shown in Figure 4. Each category was again divided based on its application domains, namely, CAV and other (non-CAV) domains. As inferred from the chart, the majority of screened papers focus on the CAV domain across various categories. However, a divergence is observed in the latency teleoperation and mitigation methods categories. In these areas, the proportion of papers from other domains surpasses those directly related to CAVs, as fewer papers have addressed these topics in the literature. This disparity and scarcity of studies reflect the importance of further research in this direction.

## 3. Teleoperation Challenges

CAV teleoperation or remote driving has a distinctive set of challenges. CAVs heavily rely on multiple sensors and communication networks, unlike mobile robots or other vehicles. Since CAVs are connected to networks, they face increased cyber threats. Further, as CAVs are expected to operate in the real world, they can be dynamically operated, over long distances and with a full range of speed environments [3,7]. They are also expected to make safe and ethical decisions. Hence, they require a robust, reliable and seamless communication system.

Communication systems can be compromised by network latency during CAV teleoperation. It is widely acknowledged in the literature that network latency is one of the major challenges of teleoperation, which can cause significant disruptions in communication systems and performance [1,3,7,8,9,10,12]. Long and variable latency poses an even more significant challenge, making it nearly impossible to operate effectively. Even today, it is an unsolved problem.

Latency can affect the quality of feedback from sensory data (visual, audio, and haptic) of the vehicle to the teleoperation station, making it difficult for the operator to acquire the current state and environment of the vehicle. Simultaneously, it affects the control command data from the station to the vehicle, in turn degrading the overall teleoperation performance in the control loop. Certain studies have indicated that latency can influence the decision making, behaviour, and mental workload of remote drivers, causing over- and under-steering of the remote vehicle and leading them to irregularly adjust their pressure on the acceleration and brake pedals [7,8,15], which thereby increases the driver’s anger and frustration [17]. Latency simultaneously impacts the vehicle in terms of energy consumption, motion sickness, and degrading the comfort of passengers [15].

It has been hypothesised in the literature that latency would have detrimental effects on the performance of both teleoperation and operator perception [18]. The performance will only decrease with increasing latency. Different types of latency (from various sources) can distinctly impact performance and adaptation strategies based on individuals [18]. For example, the effect of latency will be larger on complex system tasks than on simple ones [19]. Further, the level of automation can improve the system’s ability to tackle latency, thereby increasing the trust in the system.

Another major challenge of teleoperation is situational awareness limitations [7,8]. Remote operators may need added data to improve their perception, including viewpoint shifting and adjustment, map merging, depth, spatial awareness, and multi-sensory feedback. All these data need a good system design and interface to avoid a cognitive workload for remote operators, as it can trigger confusion and distractions that can lead to accidents.

There are also other teleoperation challenges, such as lack of physical sensing (e.g., force, sound), requiring improvement in the user interface with sensor fusion, bandwidth constraints, control issues such as lack of model estimation, uncertainties, and reliable local autonomy, and other issues related to human factors and workload requirements [7,8].

## 4. Impact of Network Latency on Teleoperation

### 4.1. Teleoperation System Model

**Figure 5:**
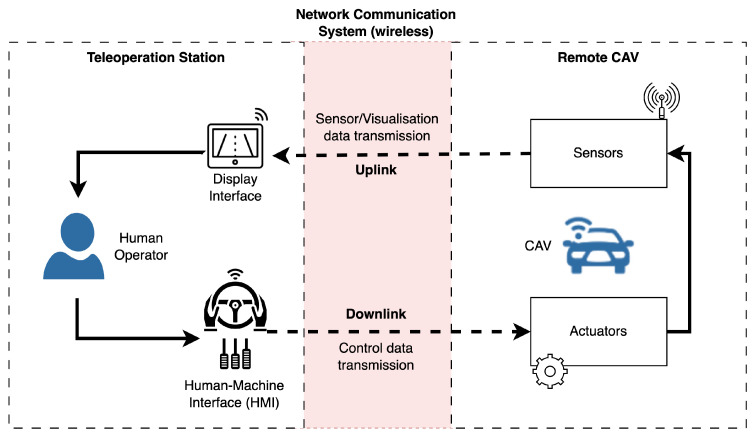
System architecture of CAV teleoperation control loop.

An overview of the CAV teleoperation system architecture is shown in Figure 5. The system architecture for the teleoperation of CAVs includes (1) a teleoperation station with a suitable human–machine interface (HMI) or control interface, (2) a remote CAV, and (3) a wireless communication network [10]. The communication network is responsible for seamless data transmission and operational efficiency between the vehicle and the station. The operated CAV transmits perception data streams obtained from sensors (e.g., video data from the cameras) to the teleoperation station via onboard communication networks for “uplink” transmission to a remote operator. The remote operator receives the visual and other perception data, and then, sends back the control data (e.g., steering and braking commands) using a teleoperation controller/interface through the “downlink” of the same communication network.

### 4.2. Wireless Technologies Used in Teleoperation

Wireless technologies play a pivotal role in teleoperation systems by enabling seamless control and communication between the operator and the teleoperation station. It is crucial to maintain the safety and security of the teleoperation communication systems, as these systems should be uninterrupted and transmit data in real-time [9]. The wireless technologies used in teleoperation with their performance and latency requirements are listed in Table 2. These are technologies that have been used across various domains. The technology performance is determined by various network parameters, including frequency, bandwidth, coverage, data rate, throughput, and latency. The most influential factors for a general teleoperation use case include latency, throughput (user data rate), data rate (maximum capacity), and coverage. Hence, only these parameters are mentioned in the table.

There has been a drastic evolution in network technologies over time, with each iteration achieving improved performance and unlocking new applications, in turn, decreasing the effects of latency. A standout example is the evolution of cellular networks, which have revolutionised the way we communicate and access data. They have evolved from first-generation 1G (from 1980) to fifth-generation 5G (from 2020 onwards) networks, where each generation is an improvement on the previous one [20], providing better coverage, higher frequencies, faster data transfers, lower latency, higher reliability, and mobility, among other improvements.

The main factors for network data traffic growth are increasing populations with smart devices, applications, and sensors, leading to the Internet of Everything (IoE) [20]. The statistics of the International Telecommunication Union (ITU) state that just global cellular traffic will rise to 607 exabytes (EBs) per month by 2025 and 5016 EB by 2030 [20]. With the continuous demand for network traffic and emerging applications (e.g., CAVs), the current technologies will have difficulty sustaining the requirements.

Latency can cause disruptions to applications relying on communication technologies. Different technologies for teleoperation exhibit various levels of latency based on their QoS factors. For example, experimental results have shown that 3G networks have a mean latency of 217 ms and 205 ms for vehicle control and video streaming, respectively. Whereas the next generation showed a slight improvement, with 4G networks having a mean latency of 183 ms for video streaming and 107.2 to 110.3 ms for vehicle controls [21]. The present and future generations of networks, including 5G and sixth generation, are expected to have much lower latency and better network quality (e.g., high frequencies, fast data transfers, high reliability, mobility) [20], satisfying the requirements and leading to efficient teleoperations. For example, teleoperation using 5G networks can have latency as low as 60 ms but as high as 260 ms in some cases [22].

As inferred from the table, the wireless technologies used in teleoperation can be broadly classified as cellular and non-cellular. Cellular technologies, especially the recent advances, provide reliable and high-speed connectivity over large geographical areas. Many studies that have used 3G, 4G, and 5G networks for teleoperation have demonstrated that latency and bandwidth requirements could be met for teleoperation applications, particularly for CAVs and remote scenarios requiring long-distance operations. In such scenarios, wired connections are infeasible. However, they have limitations in extremely remote areas such as rural areas or due to heavy traffic and congestion in urban areas, which can lead to potential latency or signal degradation. Some studies have revealed that there is an evident difference between 4G and 5G networks for teleoperation [23].

The 4G networks are just able to support the necessary infrastructure for basic remote driving capabilities [10]. The relatively high data rates and improved reliability over 3G allow sufficient quality of video and control transmission, which is essential for this use case. However, as seen in the table, latency of 100 ms can pose challenges for real-time responsiveness, leading to potential difficulty in vehicle control. Moreover, the bandwidth limitations can restrict the parallel processing and amount of sensor data. Despite these constraints, 4G serves as a standard for demonstrating the feasibility of remote driving.

Going one step further, 5G networks significantly enhance remote driving capabilities with their ultra-low latency (on the order of 10 ms), high data rates (up to 10 Gbps), and significantly improved reliability [10,24]. They offer large volumes of data that can be quickly and reliably transmitted. These features are satisfactory for various autonomous applications, including multiple high-resolution sensor data, responsive control, logistics, and emergency services [3]. They can even ensure reliable management of fleets of remotely operated vehicles.

The non-cellular classification includes diverse technologies that are more suitable for applications other than CAV teleoperation. Technologies such as WiFi, Bluetooth, and Zigbee are commonly used for short-range communications with overall good network performance. These are well suited for applications in controlled and confined environments, as in robotics. Satellite communication performs excellently in areas where other traditional terrestrial networks are impractical, with high latency given its large coverage area. However, it is expensive and is sensitive to environmental factors.

**Table 2:** Wireless technologies used for teleoperation.

Category	Technology	Latency during Teleoperation (ms)	User Data Rate (Mbps)	Maximum Capacity	Coverage
Cellular	5G [10,20,25,26,27]	11–13	4–8	1–10 Gbps	Up to several km
	4G (LTE) [10,13,20,25,26,28,29]	100	3–8	100 Mbps– 1 Gbps	5–50 km
	3G [10,13,20,28]	121–217	Up to 3.1	3.1–14.7 Mbps	Up to 10 km
	2.5G (GPRS) [10,20]	500–1000	0.120	56–171.2 kbps	∼35 km
Non-cellular	Satellite [8]	2000	$∼10^{4}$	10 Mbps (UL), 1 Gbps (DL)	1000 s of km
	LAN/WLAN/ WiFi [8,10,13,30]	50	500	11/54/600/ 1000 Mbps	∼100 m
	Bluetooth [13,25]	34–200	2	1.5–2 Mbps	∼200 m
	Zigbee [8,25]	200	0.250	250 kbps	10–300 m

Both cellular and non-cellular technologies have been emulated/simulated for more flexible, scalable, and secure experimentation. On one hand, these experiments attempt to allow the replication of real-world networks in controlled environments without risking physical systems. On the other hand, they offer the opportunity to define parameters and effectively validate and test various teleoperation applications in different scenarios and conditions. The parameters of simulated/emulated networks depend on how the user defines them. For example, in a simulation experiment, driving control had a latency of 170 ms and flight control had 1.5 to 3 s [28]. Another example, an NS3–IP-based emulator, had a latency of 16–19 ms [10]. The simulated ROS-based virtual network adapter had 64–74 ms, Rosbridge had 270 ms and WiFi had 445 ms [8,27]. However, these networks are primarily implemented in simulations and may not always yield realistic results due to real-world networks’ inherent uncertainty and dynamic nature.

Studies have also used other communication technologies in teleoperation, including the Internet through a wired Ethernet cable, VLAN, radio link, inter-process communication (IPC) wireless network, umbilical cable, dual-tone multi-frequency (DTMF) and multimodal radio frequency (RF), VideoLAN Client (VLC), and acoustics [8,30].

### 4.3. Network Latency Characteristics

Network quality in a system (e.g., teleoperation) can be defined by its QoS and quality of experience (QoE) [31,32], also known as objective and subjective factors, respectively. The objective factors include the network performance parameters. Meanwhile, the subjective factors include user performance parameters such as user interface, video quality, driving precision, comfort level, manoeuvre security, and interruptions [32].

Network latency is a QoS component that produces a time delay when transferring data across a communication network [33,34]. Usually, it is calculated as end-to-end delay or round-trip time (RTT), which refers to the time taken for data transfer from source to destination and back again to the source. Networks with longer delays resulting in a noticeable lag have high latency. A sufficient amount significantly affects the application system performance, and more elevated levels can cause system failures.

### 4.4. End-to-End Delay

**Figure 6:**
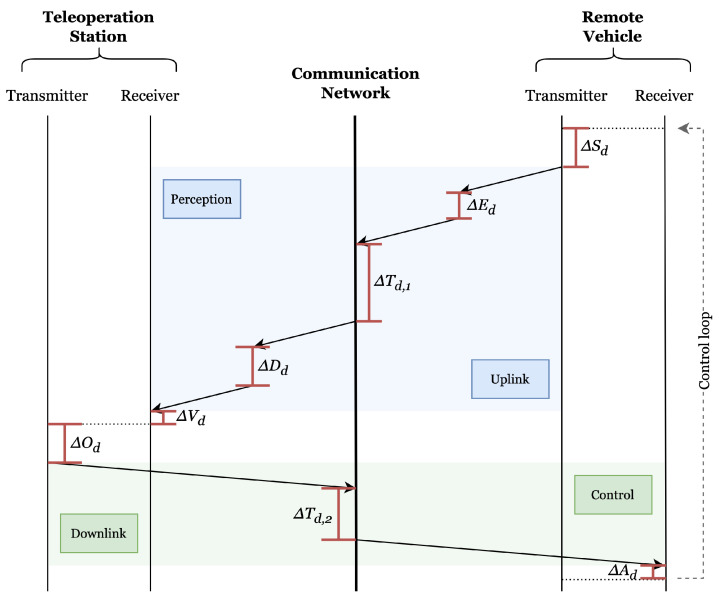
Different sources of latency in CAV teleoperation control loop. The latency in the diagram is shown by red lines, which show approximately the magnitude of each component (for illustration purposes). The blue colour region is the uplink data from sensors to the operator perception interface. Similarly, the green region is the downlink, the control data from the operator station to the vehicle actuators.

The latency sources in this teleoperation feedback loop are shown in Figure 6. Latency sources and components of CAV teleoperation in an end-to-end loop include sensor exposure delay (e.g., camera capture delay) ($\Delta S_{d}$), encoding delay ($\Delta E_{d}$), data communication delay in terms of transmission (vehicle to the station ($\Delta T_{d,1}$) and station to vehicle ($\Delta T_{d,2}$)), processing or decoding delay ($\Delta D_{d}$), visual display delay ($\Delta V_{d}$), operator response time ($\Delta O_{d}$), and vehicle actuation delay ($\Delta A_{d}$) [25,35,36]. It is challenging to mitigate all these different sources of latency in a system [15].

The one-way latency is calculated as the sum of scheduling, transmission, and re-transmission time [37]. The information might be re-transmitted if lost. The sum of delays from camera capture to display is called capture-to-display latency or glass-to-glass latency [25], which is also the one-way uplink latency. The capture-to-display latency ($\Delta_{C2D}$) is given by

(1) $$UL=\Delta_{C2D}=\Delta S_{d}+\Delta E_{d}+\Delta T_{d,1}+\Delta D_{d}+\Delta V_{d}$$

Similarly, on the one-way downlink (DL) side, control-to-response latency is the sum of delays from sending operator control commands to the vehicle actuation response. This latency also includes control command processing, encoding, and decoding delays. However, these delays are negligible when compared to delays in capture-to-display latency. Hence, they are not considered in the equation. The modified control-to-response latency ($\Delta_{C2R}$) can be represented as

(2) $$DL=\Delta_{C2R}=\Delta T_{d,2}+\Delta A_{d}$$

Finally, the overall end-to-end latency ($\Delta_{E2E}$) or RTT is the sum of delays of all the components from uplink to downlink over the network. The end-to-end latency can be represented in an equation as

(3) $$\Delta_{E2E}=UL+DL=\left(\Delta S_{d}+\Delta E_{d}+\Delta T_{d,1}+\Delta D_{d}+\Delta V_{d}\right)+\left(\Delta T_{d,2}+\Delta A_{d}\right)$$

For example, the latency values in different sources can be [25,38]:

- For camera capture, $\Delta S_{d}$ = 17–33 ms at a frame rate of 30 fps.
- For data encoding, $\Delta E_{d}$ = 17–50 ms.
- For data transmission, $\Delta T_{d,1}$ = 25–50 ms.
- For video decoding, $\Delta D_{d}$ = 17–32 ms.
- For digital visual interface (DVI) and liquid-crystal display (LCD), $\Delta V_{d}$ involves a refresh time of 10 ms, a display time of 16.7 ms at 60 fps, and a frame buffer of 17 ms.
- For joystick control command transmission, $\Delta T_{d,2}$ = 32 ms.
- For sensors and actuators, $\Delta S_{d}+\Delta A_{d}$ is roughly 50 ms.
- Network jitter is approximately below 150 ms.
- For LTE, the average handover latency is 40 ms.

Human operators have demonstrated the ability to perceive delays as low as 10 to 20 ms [28]. A latency of around 300 ms has a noticeable impact on the operator behaviour. In contrast, latencies of 1 s or more can drastically affect real-time operations. Such delays often cause operators to adjust strategies, transitioning from open-loop to closed-loop control methods, including start-and-stop or move-and-wait, sometimes even disregarding the visual feedback [18,19]. The cognitive threshold for maintaining real-time performance is typically within 0.4 s. Upon receiving feedback from the vehicle, operators must respond within 0 to 3 s, with a minimum response time of 0.2 s [39,40].

### 4.5. Modelling Network Delay

For network latency, a major problem is with the variability (unpredictable fluctuations) of the delay rather than its magnitude, which degrades the teleoperation performance [29], suggesting that there needs to be a deterministic delay [41].

Researchers have modelled delays using simulations and emulations or have gathered data from real communication networks. These delays are injected into simulated or emulated networks. The delays used in these experiments can be classified as constant (fixed time delay) or varying (incorporating network jitter) [42]. Uddin and Ryu [43] categorise some predictive methods based on constant and variable time delays. Further, the end-to-end delay can be symmetric or asymmetric [43]. Symmetric means the uplink and downlink delays are approximately equal. Meanwhile, asymmetric delays refer to different delay values in the uplink and downlink, which is usually the case for teleoperation, i.e., the uplink sensor data delays are greater than the downlink control command delays.

Many studies in the literature predominantly perform teleoperation in fixed or constant time delay settings, testing their methodologies on different levels of constant delays. For example, Sato et al. [36] use constant delay values of 150 ms, 200 ms, and 400 ms in WiFi LAN communication. Chen et al. [44] set their delay as 0.5 ± 0.1 s. Zheng et al. [45] set the control delay to 300 ms and the sensor delay to 600 ms. Moniruzzaman et al. [46,47] utilise Simulink blocks to add delays of 300 ms into their simulator model. Other similar examples include Refs. [21,22,48,49,50,51,52,53,54,55,56,57,58,59,60,61].

However, it is crucial to evaluate teleoperation performance on delay values close to real-world networks exhibiting many fluctuations. Studies that model real-world network delays tend to have varying delays that can fit a predefined time sequence (such as a distribution or a function) or model them as a random process [42]. It has been shown that the generalised extreme value (GEV) distribution can model delays for mobile communication data [35,42]. The GEV distribution has been used to model variable delay in various studies [35,42,62,63,64]. The probability density function (PDF) of GEV(ξ,μ,σ) is defined as

(4) $$f_{\xi \neq 0,\mu ,\sigma }=\frac{1}{\sigma }{\left(1+\xi \frac{x-\mu }{\sigma }\right)}^{-\frac{1}{\xi }-1}e^{-{\left(1+\xi \frac{x-\mu }{\sigma }\right)}^{-\frac{1}{\xi }}}$$

where *x* is a random variable for the delay value, ξ is the shape parameter, μ is the location parameter, and σ is the scale parameter. Based on extreme value theory, if ξ>0, the distribution has a heavy tail with $\mu -\frac{\sigma }{\xi }$ as the lower bound.

The authors in Perez et al. [65] characterise the function of RTT delays as a defined delay threshold (where it is not very significant), rapidly and linearly decaying, and then, ending with a long tail. Examples of such functions include piecewise linear [66], algebraic, logistic, and log-logistic [67]. Thus, Perez et al. model delays using the following algebraic function:

(5) $$I_{D}=\left\{\begin{array}{cc} 1-\frac{1}{2}\left\{{\left(1+x^{6}\right)}^{\frac{1}{6}}-3{\left(1+{\left(\frac{x}{3}\right)}^{6}\right)}^{\frac{1}{6}}+2\right\}, & \mathrm{if}\;x\geq 0\\ 1, & Otherwise \end{array}\right\}$$

where

(6) $$x=log_{2}\left(\frac{T}{T_{m}}\right)$$

where *T* is the interaction lag and $T_{m}$ is the model parameter.

In another example, Zhou et al. [68] use variable time delays calculated by combining constant terms for the main delay with periodic trigonometric functions of different periods for small delay variations together with normally distributed noise, as shown below:

(7) $$D=A_{0}+A_{1}sin\left(\frac{2\pi }{T_{1}}t+\phi_{1}\right)+A_{2}sin\left(\frac{2\pi }{T_{2}}t+\phi_{2}\right)+A_{3}\omega \left(0,1\right)$$

where $T_{1}$ and $T_{2}$ are periods of trigonometric terms and ω(0,1) is normally distributed noise.

Likewise, Zhou et al. [69] model delays as a sine wave distribution. Bacha et al. [70] use a random time delay based on a Gaussian distribution in the range [0.1, 1] s. Hatori and Uchimura [71] and Nagakura et al. [72] vary the delay between 3.5 and 4.0 s and 3.0 and 4.0 s. Other studies that implemented time-varying delays include [73,74,75,76,77,78,79,80,81,82,83,84].

Some studies have also considered collecting and using real-world network data for teleoperation. Zheng et al. [42] measure the delay between Michigan and California. Guo et al. [85] collect variable delay from real network data using the User Datagram Protocol (UDP). Saparia et al. [86] collect data from 4G LTE networks. Kebria et al. [87] use the Internet to collect delay data between Australia and Scotland. Sridhar et al. [88] experiment on a physical network using Amazon Web Services for Uttar Pradesh and Karnataka. Other studies that used real networks include [4,29,89,90,91].

### 4.6. Interdependency between Network Parameters and Latency

Certain network objective factors influence network latency and teleoperation [18,22,29,31,32,33,34,41,60,65,89,90,91,92]. Latency is directly impacted by distance, vehicle density, handover between cells, speed, mobility, and packet loss. Latency increases as the remote vehicle is farther away from the teleoperation station, which increases the overall RTT [32,34,90]. Wireless networks have limited bandwidth, and increased network traffic from vehicle density can result in congestion, causing delays in data transmission and increasing latency [32,91]. Increasing handover between cells might lower the throughput and increase the latency [90]. Teleoperation at higher speed demands even faster reaction times, and higher mobility also decreases the operator situation awareness [22,60,65]. Latency measures the delay in a packet’s arrival at the destination, and more latency leads to fewer packets arriving per unit of time, which might lead to higher packet loss [33].

Meanwhile, some parameters may have indirect relationships, such as bandwidth, throughput, signal strength, data rate, and coverage. For instance, although less bandwidth increases latency during peak usage, more bandwidth does not necessarily mean more data, particularly if latency is high [33,34]. A low-latency and -bandwidth network indicates that throughput will also be low. With a large bandwidth and low latency, throughput will be higher, and network connection will be more efficient [33,34,90]. Latency becomes lower with better signal strength. However, it is not a linear correlation. Signal strength may influence the throughput but not the latency [89,90]. As long as the signal strength is not too poor, the network latency is maintained within the acceptable limits for teleoperation. Higher data rates typically lead to lower latency, as more data can be transmitted and processed quickly and vice versa [32,91]. Coverage can be less in rural areas than in urban, which can lead to more latency, indicating that latency depends on location as well [32].

Some studies have shown that latency has a more significant effect on teleoperation than actuator delay [18]. Moreover, latency has a significant impact on video streaming methods such as field of view (FOV), orientation, camera viewpoint, depth perception, video quality, and frame rate [22], which might affect the situational awareness of the operator.

Further, latency depends on the wireless technologies being used. Different network types and mediums provide different QoS and have diverse requirements and effects on latency [90].

### 4.7. Network Delay Impact on Various Teleoperation Domains

Teleoperation is generally used as a fallback solution to maintain the safety of a vehicle in cases of failure scenarios such as malfunction, challenging weather, confusing situations, collision, or other such situations [93]. Other use cases of teleoperation include places where it is dangerous or difficult for humans to reach, such as telerobotics for space and underwater exploration, satellite communications, use of unmanned aerial vehicles (UAVs) and unmanned ground vehicles (UGVs) for reconnaissance missions, search and rescue, or surveillance in military/defensive applications, and also for telesurgery in medical domains [40]. The impact of network latency and requirements across various domains are detailed in Table 3.

Several studies have explored teleoperation as a critical use case within their respective domains. Although teleoperation is used in different critical applications, most studies employ robots as their teleoperation system. Some use mobile robots, while others require single- or dual-arm manipulator robots (such as telesurgery). Nevertheless, a consistent finding across these studies is the detrimental impact of latency on teleoperation performance and operational safety, ranging from compromised mission effectiveness to increased mortality risks in critical applications such as telesurgery. It is universally acknowledged that having lower latency is the best case for effective operation. The minimum acceptable latency threshold is shown to be influenced by factors such as required data rates, teleoperation distance, and, most importantly, the choice of wireless technologies.

Even within domains seemingly disparate from traditional teleoperation, such as gaming, parallels can be drawn with teleoperation regarding identical setups and the importance of responsive interactions from the operators. Thus, while the specifics and unique demands may vary across domains, the overarching concern remains the mitigation or compensation of latency to ensure optimal teleoperation performance and safety.

### 4.8. CAV Teleoperation Requirements

Considering various domains, most of the literature on teleoperation uses mobile robots with or without manipulators. Comparatively, there is less research conducted on CAV teleoperation [8,12]. Despite the similarities of challenges faced by teleoperated robots and CAVs, the latter operate in significantly more complex environments, encountering far greater challenges [7]. For example, CAVs have higher stakes and operate in adverse weather conditions, long distances from remote stations, at higher speeds, and involve ethical decision making. Hence, more research is recommended in this area.

The network requirements of CAVs are different from those of teleoperation in other domains. For example, compared to robotic teleoperations, CAVs involve realistic, uncertain, long-distance environments with usually higher vehicle speeds. The requirements of CAV teleoperation are shown in Table 4. Most studies provided requirements for latency thresholds and data rates, as the most critical QoS parameters for CAV teleoperation are the uplink data rates and the downlink latency [100].

The effect of latency thresholds can be classified as less impact, acceptable, difficult, and impossible thresholds. As mentioned earlier, using different wireless technologies for teleoperation can induce different latency thresholds. Summarising Table 4, it is evident that the downlink latency threshold is relatively lower than the uplink. By combining all the overall latency thresholds, it can be concluded that less than 170 ms has less impact on performance, and between 250 ms and 300 ms has an acceptable effect, with the average uplink latency in the range of 50 to 120 ms and downlink latency in the range of 20 to 80 ms (with some outliers). As the latency becomes higher, such as greater than 300 ms, the operator performance seems to degrade, making it difficult. Further degradation is seen above 700 ms and 1 s, making teleoperating almost impossible.

Similarly, regarding data rate, it can be inferred that CAVs have significantly higher uplink data needs than downlink needs. This is because CAV sensor perception (such as camera, lidar, and radar sensors) takes up more data than the control commands. The average uplink data rate can range from 8 to 50 megabits per second (Mbps), as camera data themselves take about 8 Mbps, while data rates for the downlink are in the range of 0.25 to 5 Mbps.

Correspondingly, there are network QoS and other environmental requirements for CAV teleoperation not covered in Table 4, such as a bandwidth of about 30 to 100 MHz, depending on urban or rural environments, a throughput greater than 3 Mbps [65], visual quality with a minimum or average resolution of 640 × 480 along with approximately $150^{\circ }$ view angle FOV, and a fluent image of 25 to 30 frames per second (FPS) is sufficient for teleoperators [90]. Further, the service reliability is preferred to be 99% for uplink and 99.999% for downlink [32,91]. Suppose the teleoperation service is scaled up in the coming years. In that case, it will be feasible for the current networks to handle a vehicle density of about 10 vehicles per kilometre square (veh/km^2^) [91]. Most studies focus on driving at low speeds, such as less than 50 km/h [32]. However, a few studies suggest the maximum speed teleoperated vehicles can tolerate is 100 to 250 km/h, above which performance will degrade significantly [41]. Therefore, to avoid such degradation, it is recommended to perform remote driving at low-to-medium speeds rather than at high speeds. With regards to distance, CAV teleoperations can be conducted over long distances, for example, 5200 km in [65] and 19,000 km in [90].

## 5. Latency Mitigation Strategies

Most delay compensation or mitigation approaches can be broadly classified into control, perception, and network optimisation methods. These methods are reviewed further in the following subsections. However, it is worth noting that, in this paper, it is assumed that the network optimisation methods are already performed. Hence, they are covered very briefly. This paper primarily focuses on the control and perception approaches. A comprehensive list of latency mitigation strategies for control and perception is provided in Table 5 and Table 6, respectively. A practical summary of these strategies highlighting their advantages and disadvantages is later provided in Table 7.

**Figure 7:**
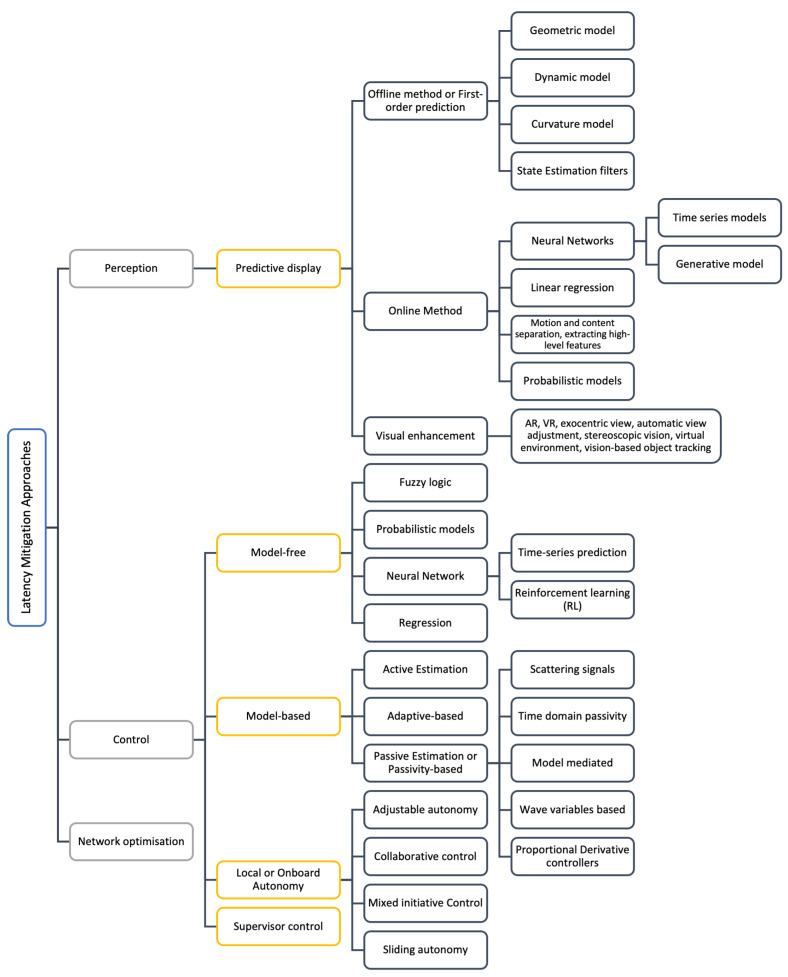
Latency mitigation strategy classification.

Predictive methods have been shown to be well suited for delay compensation on both the downlink and uplink of teleoperation [70,113]. In the realm of predictive methods, passivity-based and predictive display-based methods have been most commonly used. They have been shown to reduce inconsistency. However, the performance degrades with increasing and variable time delays. A broad classification of latency mitigation methods is demonstrated in Figure 7.

### 5.1. Control Latency Approaches

Control latency is the delay in data transmission of control commands from the operator to the vehicle (i.e., on the downlink). A list of latency mitigation strategies for control is provided in Table 5. On the one hand, earlier researchers focused on mathematical models for accurately predicting and compensating for undelayed controller signals [45]. These methods are well known to be model-based approaches [69]. They are usually modelled according to control theorems, out of which the most extensively researched compensation methods are based on the perspective of passivity theory [44,51,114]. Passivity-based control methods predict the signals after the delay without knowledge of the magnitude of the delay. They ensure the stability and transparency of the bilateral teleoperation systems. A few examples include wave variable-based methods, scattering signals, time-domain passivity analysis (TDPA)-based methods [48,77,79,80], and model-mediated teleoperation (MMT) [69,80,115]. The major drawback of these methods is that they require knowledge of system dynamics. Moreover, their performance degrades due to model errors and complicated conditions with variable time delay, large disturbances, or extensions to multilateral systems [69].

On the other hand, model-free approaches based on data-driven models have been developed. These methods have grown popular as they are more robust and do not require any knowledge of system dynamics [69]. They are adaptable and can deal with disturbances and uncertainties while making predictions. A popular example is neural networks (NNs) [69,116]. Time-series prediction methods effectively mitigate the effects of control latency by predicting or imitating the operator behaviour [12]. They tend to be more adaptable to non-linear data. In the literature, most studies employ or combine traditional NN methods such as auto-encoder (AE), variational auto-encoder (VAE), recurrent neural network (RNN), and long short-term memory (LSTM) for time-series data [12]. Among these, RNN [74,117] and LSTM [55,69,74,118,119] are more suitable for time-series prediction. However, sometimes RNNs cannot learn long-term dependencies, whereas LSTMs have the advantage of retaining both long- and short-term memory. A limitation of LSTMs is that they are incompatible with dynamic output lengths and may require retraining [12]. More recent methods, for instance, sequence-to-sequence (Seq2Seq) models [120] and generative adversarial networks (GANs) [121] have shown great performance for time-series prediction, and they can tackle these limitations. GANs learn the distributions of time series well and also adapt to new data, but might not capture dependencies. Meanwhile, Seq2Seq has a better mapping of input and output data relationships.

Other significant control prediction methods include state estimation filters, motion models, linear regression, proportional–derivative models, statistical models, and probabilistic models [116], among others. Linear regression is a simple approach with good accuracy for smooth and linear motion. However, it is prone to errors and overshooting [122]. State estimation filters, such as Kalman filters (and their extensions), are commonly followed by motion models and are robust against fluctuations [122]. However, they are sensitive to noise and computationally expensive. Proportional–derivative methods achieve stability without knowing system dynamics, but their robustness may come at the cost of transparency [45]. Statistical models represent the control loop in the form of equations; for example, auto-regressive models, moving average models, and their extensions. However, these methods are unsuitable for non-linear and dynamic data, which can be expected in the real world [12].

Other control approaches aim to minimise delay, including supervisor control, control autonomy augmented reality [113], and adaptive-based control [70,113]. Approaches such as supervisor control involve the operator sending high-level commands, minimising communication requirements and delay, where the remote robot or vehicle needs planning and control algorithms. However, it does not involve continuous teleoperation [114]. Onboard control autonomy capabilities can be activated to mitigate the effects of delay. Such approaches can be classified as adjustable autonomy, collaborative control, mixed-initiative control, and sliding autonomy [51]. They are all impacted by communication delays and are computationally expensive [45].

**Table 5:** Mitigation strategies for control latency.

Reference	Domain	Teleoperation Entity	Algorithm
[45]	Vehicle teleoperation	Control delay and sensor delay	Model free predictive framework
[69]	Robotic teleoperation	Delay and control prediction	LSTM-based bilateral active estimation model (BAEM)
[70]	Telesurgery, control framework	Control and force feedback	Kalman filter and RL-based DDPG algorithm
[49]	Robotic teleoperation	Control, steering	PD controller
[42]	Teleoperated military UGVs	Control, steering, and heading prediction	Steering-model-based feedforward predictor, model-free
[50]	Robotic teleoperation	Control and haptic data	Input-to-state stability (ISS) controller
[51]	Robotic teleoperation	Control and haptic	Llewellyn’s criterion and a passivity-based criterion with and without the wave transformation.
[123]	Robotic teleoperation (medical)	Control prediction	Gated recurrent units (GRUs) integrated with a double deep Q-learning network (DDQN) algorithm
[87]	Robotic teleoperation	Control	Adaptive interval type-2 fuzzy neural network, Lyapunov–Krasovskii method
[124]	Space robotic teleoperation	Control interface	Interactive planning and supervised execution (IPSE) teleoperation system
[115]	Robotic teleoperation	Control uncertainty	Integrate RL with model-mediated teleoperation (MMT)
[74]	Telepresence robot	Predict control commands	Integrate RNN and LSTM with RL-DDPG for predicting the behaviour of the teleoperator
[44]	Teleoperation of multiple robots	Control	A wave-variable-based time-delay-compensated four-channel architecture for multilateral teleoperation
[114]	Bilateral teleoperation	Control	Adaptive NN based on Markov jump, partial feedback linearisation using nominal dynamics, Lyapunov–Krasovskii functional
[54]	Robotic teleoperation	Control—handwritten letter drawing	K-means, Gaussian mixture model (GMM), hidden semi-Markov models (HSMMs), and linear quadratic tracking (LQT) for motion recognition and segmentation
[48]	Bilateral robot teleoperation	Control and force feedback	Force controller and time-domain passivity approach (TDPA)
[125]	Bilateral teleoperation	Improve control	Integral-order and fractional-order PD controller, along with frequency-domain analysis for maximum upper bound of delay, Lyapunov–Krasovskii functional
[118]	Train to ground communication	State and network prediction	LSTM and high-degree polynomial linear regression (HPLR)
[53]	UGV teleoperation	Predict human steering behaviour	Two-point visual steering model based on PI controller, adaptive control of thought–rational (ACT-R) cognitive model
[75]	Teleoperating autonomous vehicles, flight simulator	Control prediction	PID control model to predicted motion
[76]	Robotic teleoperation for surgery	Motion scaling for control	Constant, position and velocity scaling to improve performance and decrease errors during delay
[72]	Tele-driving	Control prediction	Model predictive control (MPC) and Kalman filter
[77]	Robotic bilateral teleoperation	Control	Radial basis function (RBF) neural network (NN)-based four-channel wave-based time-domain passivity approach (TDPA), Lyapunov control
[71]	Robotic teleoperation	Control prediction	Model predictive control (MPC) and linear interpolation to predict state and avoid obstacles
[78]	Connected vehicles	Connected cruise control	Optimal control using linear quadratic regulation and minimising a cost function
[79]	Bilateral teleoperation	Control	TDPA and time delay power network (TDPN) to achieve position synchronisation
[55]	Teleoperated ground vehicles	Control prediction	Predicted trajectory guidance control (PTGC) and deep-learning-based LSTM model to predict trajectory
[43]	Bilateral teleoperation	Control prediction	Survey of predictive control approaches
[126]	Road vehicle teleoperation	Control and haptic prediction	Two-stage predictive approach environment model (Bayesian filters) and haptic feedback to compensate delay, warn collisions and assists
[80]	Bilateral teleoperation	Adaptive control, reduce haptic data during delay and improve QoE	Delay-adaptive control switching scheme between TDPA and MMT
[81]	Bilateral teleoperation	Adaptive control	Radial basis function (RBF) neural network-based controller
[82]	Bilateral teleoperation	Adaptive control, position tracking and force feedback	Radial basis function neural network (RBFNN)-based adaptive sliding mode controller and projection mapping by saturation function
[127]	Robotic teleoperation	Control sensory manipulation, haptic feedback	Augmented sensory manipulation based on motor learning and rehabilitation principles, inverse kinematics for human adaptation during delays
[83]	Tele-driving	Control	Model predictive control (MPC) and improvement in cost function
[84]	Tele-driving	Control	Model predictive control (MPC) and model error compensator (MEC), Kalman filter
[128]	Telepresence robot, IoT, healthcare	Delayed control signals	Markov model, deep reinforcement learning (DRL)-based deep deterministic policy gradient (DDPG)
[12]	Robotic teleoperation	Control prediction	Survey of time-series prediction: Statistical approaches and neural network approaches
[88]	Robotic teleoperation	Control	Model-free predictor modified with adaptively varying predictor parameter
[58]	Strategy games	Control prediction	Artificial neural networks with internal states predicting future position of mouse
[64]	Vehicle teleoperation	Control and steering prediction	Non-linear model predictive control (NMPC), successive reference-pose tracking (SRPT) to predict and improve path tracking, reference pose and speed

### 5.2. Perception Latency Approaches

Perception latency is the delay in the perception system at the remote operator’s end. A list of latency mitigation strategies for perception is provided in Table 6. Predictive displays generate the probable vehicle response on the delayed or predicted view based on the current actions of the operator. They have been shown to effectively withstand and compensate for the negative impacts of latency during teleoperation [42]. These methods can be classified as offline or online [129]. The former involves using prior knowledge of static environments and operator behaviour models before making predictions. The latter is suitable for dynamic environments and makes near real-time predictions to provide instantaneous feedback.

During remote driving, the operator can receive sensory feedback in three main forms: visual, audio, and haptic [7]. However, most studies do not focus on audio and haptic feedback. Therefore, visual data are significant for the remote operator. Predictive display methods mainly focus on visual feedback prediction. However, most of the research on predictive displays uses simulation-based experiments [36].

Earlier offline approaches estimate and predict images that rely on first-order prediction techniques, depending on the system, delays, and actions of the operator [45]. They are restricted to state prediction and future pose estimation. For example, estimating the visual impact of control commands, heading angles, and steering wheel angles, predicting position and trajectory, or by using other dynamic, curvature, and geometric models [52,129]. However, reliance on geometric models for feature extraction and matching in low-textured scenes may lead to inaccuracies [129]. Moreover, first-order techniques are ineffective for high-latency, high-speed, and long-distance teleoperations and do not include factors related to the uncertainty of future events. Integrating these first-order approaches with recent state-of-the-art approaches that use artificial intelligence (AI) and NNs is challenging [46,51].

Similar to the model-free control approaches in the previous section, the modern online prediction approaches involve deep neural networks (DNNs). They mainly involve data-driven models for time-series predictions of future-perspective video frames [8]. These approaches have been shown to give good results in terms of performance. The advanced methods for predictive display include the use of generative AI methods for pixel synthesis (e.g., GANs) [46,129,130], pixel transformation and time-series methods (e.g., LSTMs) [61], and probabilistic models [8]. Alternate methods that reduce the burden on computation and the interface can be considered, such as motion and content separation and extracting higher-level features in the visual feedback. Though DNNs offer accurate predictions for non-linear and long-term signals, they require large amounts of data to train for this accuracy, which can be costly in terms of computation, memory, and time [15]. Additionally, they are considered black-box models, which affects their transparency and trustworthiness, especially in safety-critical applications such as remote driving. Recent studies have also demonstrated that NNs’ predictions can be altered/manipulated by pixel adversarial attacks [131,132].

Other visual feedback enhancement techniques for enhancing teleoperators’ overall situational awareness (and not for delay compensation) include exocentric view, automatic view adjustment, stereoscopic vision, virtual environment, vision-based object tracking, and predictive systems [51].

**Table 6:** Mitigation strategies for perception latency.

Reference	Domain	Teleoperation Entity	Algorithm
[133]	CAV teleoperation	Video stream, encoding latency	Image processing approaches, video encoder configuration
[122]	Telepresence	Head-mounted display (HMD) prediction	Weighted least squares, Kalman filter, weighted sum
[129]	Robotic teleoperation	Predictive display	CycleGAN
[116]	Tactile Internet-based remote robotic surgery	Predicting haptic commands	Gaussian process regression (GPR)
[47]	Robotic teleoperation	Video transformation enhancement	Two video transformation-based assistive visual interfaces
[36]	CAV teleoperation	Visual, predictive display for latency	Geometric model, homographic transformation
[52]	CAV teleoperation	Predictive display	Predicted vehicle state, curvature model on steering angle
[46]	UGV robot teleoperation	Predictive display	Integrate Pix2Pix conditional GAN with optical flow
[39]	Robotic teleoperation	Predictive display and control	Kinematic model for augmented predictive display (for state and trajectory) and autonomy of high-level control commands
[86]	Tele-driving vehicle	Visual and control	Model predictive control (MPC)—kinematic bicycle model and potential fields, augmented reality, predictive display
[61]	Telerobotic surgeries during military operations	Visual and control	Virtual representation with object recognition (Mask RCNN semantic segmentation, Kalman filter state estimation) and alpha-blended layout. High-level surgical actions using history from LSTM
[63]	Vehicle teleoperation	Predictive display	Vehicle position and perspective-predictive image transformation
[56]	UGV teleoperation	Predictive display	Image transformation and state estimator for feedforward and feedback functions to estimate the vehicle position
[35]	Vehicle teleoperation	Predictive display	Image transformation, perspective projection, and Smith predictor
[57]	Road vehicle teleoperation	Predictive display	Grid-based distribution, TV-L1 optical flow, semi-global matching (SGM)
[134]	Video streaming	Video rate control during latency	Markov decision process, greedy approach, and stochastic gradient descent (SGD)
[8]	Robotic teleoperation	Visual, control, and other entities	Review enhancement techniques
[15]	Robotic and CAV teleoperation	Visual and control	Review some mitigation techniques
[130]	Robotic telesurgery	Predictive display and control position	Pix2Pix conditional generative adversarial network (cGAN) for predicting tool position during surgery

### 5.3. Network Optimisation Approaches

These optimisation methods target the latency during data transmission at the network level. Parvez et al. [135] provide a survey on latency reduction solutions for 5G networks. They divide the approaches into radio access network (RAN) solutions (e.g., short frame/packets, new waveform designs, mmWave aggregation), core network solutions (e.g., high-speed backhaul, mobile edge computing (MEC)/fog-network architectures), and caching solutions (e.g., distributed, centralised caching).

Various approaches have been proposed to optimise the network for reduced latency. For instance, Hollinghurst et al. [136] use redundant messages to exploit randomness across multiple paths, leading to low latency. Similarly, Hui et al. [137] consider a redundancy-aware federated learning architecture for efficient and cooperative vehicular networking and improved data quality accuracy. Ndikumana et al. [138] propose intelligent infotainment caching models in autonomous vehicles, where they use deep learning to predict contents that need to be cached. Their method can minimise content downloading delays. Belogolovy et al. [38] reduce latency by using multiple links, enabling rate control and scheduling in combination with slice-by-slice video processing. Hui et al. [139] develop a smart-contract-based secure edge computing architecture, which offers low-cost services for vehicles in 6G networks. Heryana et al. [140] focus on reducing video streaming latency in vehicle teleoperation through network factors. They apply UDP and the Real-Time Messaging Protocol (RTMP), tune the encoder, and further apply data compression. In another representative work, Zhang et al. [141] utilise DetNet for telesurgery, integrating time-sensitive networking (TSN), software-defined network (SDN), and other technical features for a more deterministic network with low latency and jitter. Qiong et al. [142] propose deep Q-learning to predict the optimal minimum contention window (MCW) for improved vehicular communication and age fairness. Kousaridas et al. [100] analyse the requirements of QoS predictions and discuss the architecture of a prediction model for 5G V2X applications, specifically for remote driving use cases. Further to that, Barmpounakis et al. [143] use LSTMs for QoS prediction of 5G networks for CAVs.

**Table 7:** Summary of mitigation strategies with pros and cons [12,15,123,144].

Algorithms		Applicability	Pros	Cons
Offline methods or first-order prediction		Perception	Simple and easy to implement	Restricted to state prediction and pose estimation. Heavily depends on system model. Ineffective for high-latency, high-speed, and long-distance teleoperation
Neural network	Time-series models	Both	Long-term dependencies	Prone to overfitting and require memory, require sequential data
	Image segmentation	Perception	Targeted instance or semantic information	Computational complexity and require data reliability
	Generative model	Perception	Adaptive to new data and learns distribution of time series	Cannot guarantee to capture dependencies
	Reinforcement learning	Control	Self-learning capability and effective in multi-agent coordination	Computationally complex
Regression		Both	Simple, captures future trends	Sensitive to outliers, may not be generalised, overfitting
Motion and content separation, extracting high-level features		Perception	Targeted instance or semantic information	Computationally complex
Probabilistic models		Both	Provides uncertainty and likelihood	Can be complex, may not be suitable for real-time prediction
Visual enhancement		Perception	Improves situational awareness	Sensitive to noise and may not be suitable for dynamic data
MPC-based		Control	Applicable for short and long delays	It is computationally expensive
Fuzzy logic		Control	Robust to uncertainties	Less precise
Active estimation		Control	Optimal and accurate solution	Complex, computationally expensive, heavily depends on system model
Adaptive-based estimation		Control	Robust to system dynamics	Complex, computationally expensive, Heavily depends on system model
Passive estimation or passivity-based		Control	Maintains stability of system	Does not have predictive capability
Local or onboard autonomy		Control	Backup, real-time response	Affected by delay and requires computation, maintenance, and updates
Supervisor control		Control	High-level commands	No continuous commands

### 5.4. Initiatives, Standardisation Efforts, and Guidelines

Standards bodies and industry alliances play a crucial role in maintaining quality, consistency, interoperability, and reliability across industries and domains by providing formal guidelines, products, services, responsibilities, specifications, and requirements.

There are numerous standards for vehicular communications. For example, the Institute of Electrical and Electronics Engineers (IEEE) and European Telecommunications Standards Institute (ETSI) developed standards for dedicated short-range communication (DSRC) (IEEE 802.11p or IEEE 1609 based) [145,146] and cooperative intelligent transport systems (C-ITSs) [146]. The 3rd Generation Partnership Project (3GPP) develops standards for wireless networks, inclining towards cellular vehicle-to-everything (C-V2X) [145]. The IEEE developed communication protocols and standards for the Internet of Vehicles (IoV) and intelligent transport systems (ITSs) among various interfaces, including vehicle-to-vehicle (V2V), vehicle-to-infrastructure (V2I), and so on [31].

Standards for CAVs have been established by SAE standard J3016 since 2014, which has defined taxonomies on driving automation systems and levels [10,14,147], where SAE J3016 focuses on defining remote assistance and remote driving.

However, there is not a sufficient number of standards for vehicle teleoperation that provide the requirements and responsibilities of remote driving.

Recognising this gap, the BSI has developed several publicly available specifications (PASs) for the remote teleoperation of CAVs. According to BSI PAS 1886, the roles of remote operators include remote monitoring, remote assistance, and remote driving to supervise, support, and directly control the vehicle, respectively [9]. The remote operator is responsible for continuously monitoring remotely at all SAE levels, including fully automated vehicles [10]. SAE levels 0–3 require remote driving from human operators as a fallback solution to achieve MRC, sometimes including level 4. Remote assistance is primarily performed in SAE levels 3–5. Furthermore, BSI PAS 1884 includes a section on training for remote operators, ensuring the quality and proficiency of this critical role [148].

In addition to standards, some efforts from white papers and industry reports address the current state, use cases, and requirements for safe CAV teleoperation. Projects such as Endeavour-WP15B [149], 5GAA [24,106], 5GCroCo [108,109], and 5GMobix [110] have contributed insights and recommendations, particularly concerning impacts and requirements for CAV teleoperation and network latency. Moreover, the initial deliverable of the SAVOR project [150] undertakes experiments and provides recommendations on remote monitoring and teleoperation of CAVs.

Other famous initiatives from corporate companies aim to develop self-driving systems, including Openpilot Level 2 [151], Nissan’s ProPilot Level 2 [152], BMW’s Personal Co-Pilot Level 2 [153], Mercedes-Benz’s Distronic Level 2 [154] and Drive Pilot Level 3 [155], Audi A8 Level 3, Baidu Apollo Level 4 [156], Google Waymo’s World’s Most Experienced Driver Level 4 [157], Ford’s and Volkswagen’s Argo AI Level 4 [158], General Motors Level 4 [159], Uber [160], Tesla, and Mobileye [3,4,161]. Most level 2 systems rely on cameras and radar sensors, while levels 3 and 4 additionally use lidar sensors to perceive the environment. Regarding automated control, all these levels have distance keeping, lane following and keeping. The level 2 vehicles, in contrast to 3 and 4, do not have lane-changing autonomy [4].

A few of these initiatives have considered remote operations [14]. Examples include Nissan Seamless Autonomous Mobility [162], ARGO AI remote system [158], Aurora Teleassist [163], Voyage Telesisst [164], Zoox TeleGuidance [165], and UBER remote system for remote guidance and assistance. Likewise, Valeo [166] has Drive4U Remote service and Waymo level 4 ADS has human input for fleet response. Meanwhile, companies such as Bosch [167], Baidu Apollo, Einride [168], Phantom Auto [169], Ottopia [170], Fernride [171], and Vay [172] have considered remote driving solutions [14].

### 5.5. Method Correlation with CAVs

There are very few studies on delay mitigation methods for CAVs. Nevertheless, it is feasible to adapt methodologies employed in other domains to fill this gap, provided the distinctive requirements of CAVs are taken into consideration. However, it is essential to note that the developed methods should be optimised and minimise any additional latency they introduce in the teleoperation control loop [8]. A potential solution is using predictive feedback methods, such as the predictive model-free and online approaches, which are well suited for addressing both control and perception delays, respectively. The developed methods should focus on dynamically adjusting their sensor retrieval and driving behaviour based on the network conditions. One can achieve safe and reliable teleoperation even while predicting the short-term or immediate future [12], i.e., during latency, it is sufficient to predict approximately the number of frames based on the visual update rates (the cameras FPS). Further, to improve the operator situational awareness, it is also recommended to use multi-sensory feedback (e.g., cameras along with lidars, radars, audio, and haptic). However, appropriate sensor choice is critical for the sensor fusion to be safe and reliable, since camera-based sensors may require higher computational power and are sensitive to lighting conditions. Meanwhile, active sensors such as lidar and radar can be affected by interference from other sensors [173].

Moreover, most studies use simulations to experiment and test the teleoperation environments [36]. This is a cost-effective solution, yet it may not always provide realistic results due to simplistic assumptions and constrained conditions. Again, real-world experiments also have their downsides, including being expensive to build and, most importantly, not reproducible for further research.

## 6. Conclusions and Future Directions

This paper provides a systematic literature review on the impact of network latency during teleoperation in CAVs. First, the latest trends and existing challenges have been discussed, followed by a system model of teleoperation along with sources of latency, the wireless technologies in teleoperation, and the impact of network latency across domains. Performing the teleoperation of CAVs is challenging, as they operate in significantly complex and dynamic environments with high mobility over long distances. The specifics and unique demands may vary across domains, but a consistent finding is the detrimental impact of network latency on teleoperation performance.

The CAV teleoperation system has a distinctive set of requirements, including low latency, sufficient data rates, and high reliability. The most critical QoS parameters for CAV teleoperation are the uplink data rates and the downlink latency. This is because CAV sensor perception has significantly greater data needs than control commands, and a delay in control commands is more alarming than a delay in perception. For ideal CAV teleoperation, an acceptable latency threshold is preferred to be less than 250 ms or 300 ms, with the average uplink latency in the range of 50 to 120 ms and downlink latency in the 20 to 80 ms range. This threshold correlates with a few safety-critical applications such as telesurgery, UAV, nuclear, ordnance disposal, and some network games. Similarly, when it comes to data rate, the average uplink data rate can range from 8 to 50 Mbps, while data rates for downlink are in the range of 0.25 to 5 Mbps. The best wireless technology candidates to meet these needs are cellular networks, which provide high-speed communication and sufficient coverage for long-distance CAV teleoperation. As these technologies continue to evolve, from 4G to 5G and anticipated 6G, they play a crucial role in shaping the future of autonomous and remotely operated vehicles. The groundwork was laid by 4G by providing the basic infrastructure necessary for initial teleoperation capabilities, despite its limitations in latency and bandwidth. The advent of 5G significantly enhanced ultra-low latency, high data rates, and robust reliability, enabling real-time high-definition video streaming and precise remote control during teleoperation. This has made 5G the current standard for advanced remote driving applications, enhancing safety and operational efficiency. While still in the conceptual and development stage, the next generation 6G networks will be better suited to sustain emerging applications and network traffic.

Then, state-of-the-art latency mitigation and compensation strategies have been analysed. The latencies can occur in different sources of the CAV teleoperation system, including transmission delay, operator response time, encoding and decoding, visual display, sensors, and actuator delays, which makes delay mitigation quite challenging. The predictive methods are well suited for latency mitigation in both teleoperation control and perception approaches. Recent studies use model-free and online predictive approaches for control and perception latencies, respectively. Among these, the most used are deep neural networks for time-series predictions. In addition, there are also other equally capable methods involving regression, probabilistic models, and local autonomy.

Enlightened by the aforementioned analysis, this paper proposes the following future research directions:

- More empirical studies across diverse environmental settings would be beneficial in determining the true and accurate CAV requirements. This also would establish a comprehensive understanding of CAV teleoperation. It is important to consider the impact of different levels of network reliability with varying QoS parameters, particularly latencies and bandwidths. For example, the studies can include testing performance in uncertain and dynamic environments, the trade-off between video quality and control responsiveness, and quantifying the robustness of predictive algorithms.
- When developing latency mitigation methods, it would be beneficial to integrate various approaches. For example, traditional statistical methods (i.e., first-order prediction techniques) can be integrated with modern AI-based approaches to overcome their limitations. This will allow the methods to be effective and reliable against non-linear dynamic data (such as high-latency and high-speed teleoperations) without heavily relying on complex and accurate system models. Another example, integrating predictive methods with probabilistic approaches can provide the likelihood and uncertainty of predictions. This would help in determining the reliability of the developed method.
- During teleoperation system experimentation, it is recommended to replicate real-world network conditions (including communication networks and injected delays) as closely as possible, as they offer robust evaluation by extensively testing the system on time-varying and fluctuating latency. Note that teleoperation in the presence of time-varying latency is more challenging than under constant and predictable latency.
- While there are a few white papers and initiatives from industry projects and alliances for CAV teleoperation, there is a lack of sufficient standardisation in critical aspects such as network latency requirements to guarantee consistent performance evaluation, data security protocols to protect sensitive information and ensure the integrity of communication, and interoperability between different teleoperation system components. Therefore, we recommend additional standardisation efforts to provide formal guidelines, products, services, specifications, and requirements accordingly.

## Figures and Tables

**Table 1:** Comparison with other survey papers.

Other Surveys							Our Approach
**Architecture**	Teleoperation system [8,13]						Sources of latency in teleoperation system model (Section 4.1 and Section 4.4)
**Challenges**	Main teleoperation challenges [7,8]						Brief discussion of challenges of teleoperation, with a focus on CAVs and latency as a key challenge (Section 3)
**Communication Network**	A few wireless technologies [13]		Little about network requirements [10]		Impact of latency on operators [1,15]		Most wireless technologies used in teleoperation including cellular and non-cellular (Section 4.2)
**Domain**	Robotics [8,12,13]			CAVs [1,3,7,10,14,15]			Latency impact on various teleoperation domains, and then, focusing on CAVs (Section 4.7 and Section 4.8)
**Mitigation Strategies**	Control and time-series prediction models [12]			A few latency mitigation strategies [8,15]			Detailed analysis of mitigation strategies for control and perception, with a brief discussion of network optimisation (Section 5, Section 5.1, Section 5.2, Section 5.3 and Section 5.5)
**Initiatives and Standards**	Real use cases (specific to situation awareness) [1]; standardisation for road vehicles [10]; cover standards and technical reports [14]; a few industry applications [15]						Brief discussion of initiatives and standards (Section 5.4)
**Auxiliary**	Situational awareness [1,13]	Interface enhancement [7]		Driving feedback and support control [15]		Other enhancement techniques for different challenges [8]	Modelling delay for teleoperation, QoS parameters interdependency with latency (Section 4.5 and Section 4.6)

**Table 3:** Impact of network latency on teleoperation across domains.

Teleoperation Domain	Applications	Impact of Latency	Teleoperated System	Wireless Technology	Latency Thresholds	Other Requirements
Telesurgery (Medical) [61,94,95]	Robotic colorectal surgery, neurosurgery, spinal surgery, telestenting, laparoscopic surgery	Degradation in teleoperation performance leading to increased mortality risks	Single-/dual-arm robot, robotic surgical system, UAV	LAN, commercial Internet, WiFi	16–172 ms; recommended between <200 ms and <=250 ms, acceptable <330–370 ms	Data rate: 6 Mbps; distance (remote station to vehicle): 103 miles, 2848 km, 4500–8500 km.
^1^ ASUV [40,96]	Remote barge control: Transport and logistics in river/seaports, autonomous ships/barges, maritime	Can introduce significant risks of accidents	Automated barge/ship	Satellite, WiFi, 4G, 5G network	E2E: 35 ms, video feedback <22 ms and 50 ms, camera stream <22 ms and 50 ms	Data rate: 2 Mbps.
	Underwater operations: ocean science/engineering, marine science and maintenance	Can compromise critical operations and maintenance	Remotely operated vehicles with single-/dual-arm manipulators	Sonar or acoustic communication links, visual light communication (VLC), underwater radio frequency transmission	1–2 s (optical link), 1 min (acoustical link)	N/A
^2^ UAV [25]	Search and rescue, disaster situations, network coverage, construction, delivery	Can degrade real-time situational awareness and response times, compromising mission effectiveness	Remote drone	Bluetooth (IEEE 802.15.1), Zigbee (IEEE 802.15.4), WiFi (IEEE 802.11x), 5G cellular networks	<50–100 ms, no effect <144 ms and <240 ms, significant effect at 1000 ms	Data rate: 50 Mbps, control: 60–100 kbps, visual: 4 Mbps; bandwidth: 25 MHz; frame rate 20–25 Hz, 30–60 fps; height: 200 m–450 m; distance 20 km.
Nuclear [40]	Nuclear decommissioning, reactor maintenance	Can increase risk of accidents/errors	Dual-arm manipulators with force feedback and articulated boom	N/A	<=200 ms	Visual update: 5 fps.
Ordnance Disposal [40]	Bomb and mine clearance, nuclear and hazardous material handling, explosive ordnance disposal robots	Can reduce precision in handling hazardous materials, increasing the risk of accidents/detonations.	Various mobile systems with single- or dual-arm manipulators	Fibre optic connection, wireless	<=200 ms, <1 s (wireless)	N/A
Space [19,39,97]	Space robotic exploration, resources mining and extraction, debris removal	Can increase the risk of accidents and impede mission efficiency	Robotic spacecraft and rovers, include robot manipulators	Satellite communication, deep space network, radio frequency	Earth to low Earth orbit is 0.4 s at a minimum, Earth to Moon: up to 3 s, Earth to Mars: 8.6–40 or 45 min	Bandwidth: few 100 bps to 3 Mbps or greater; distance: Earth to Moon: 384,400 km, Earth to Mars about 140 million miles.
Games [98,99]	Network games, first-person shooter games, racing games, moving target selection	Can degrade player’s performance and QoE	Game controllers, TV, PC, virtual reality	Wireless networks, Internet, cloud-based	Network game: max. 120 ms, first-person shooter games: acceptable 150–180 ms, racing games <100 ms, fast targets <50 ms, slow targets <150 ms, moving targets degrade performance 40–400 ms	Frame updates: 30–60 fps; online or virtual.

^1^ ASUV—autonomous surface and underwater vehicle; ^2^ UAV—unmanned aerial vehicle.

**Table 4:** CAV teleoperation requirements.

Reference	Scenarios	Latency Thresholds	Data Rates
[101]	At low speed with a multi-camera system, low visual quality	600 ms	Up to 2 Mpbs
[65]	Commercial NSA 5G measurements in three different locations: cross-border trial site (between Portugal and Spain), test site, and laboratory	RTT minimum is 45 ms; <50 ms is infrequent; minimum of 80–100 ms	N/A
[90]	Dataset of 78 h and 5200 km of driving in different areas of Germany over LTE networks	250 ms, 300 ms impossible to control	Steering commands 0.25 Mbps; 1–3 Mbps for one stream; UL: 3 Mbps and DL: 0.25 Mbps
[102]	Multi-operator switching to improve LTE coverage	100 ms	UL: 3 Mbps
[22]	Lane changes, sharp turns, lead vehicle brake, curve, roadblock, highway	Three studies—delays of up to 300 ms, other studies 400–3000 m; avg. 120.3 ms in WLAN; median of ∼55.14 ms; 96% delays 250 ms; 5G 60–260 ms	N/A
[93]	Real-time streaming on LTE	DL median 100 ms streaming frames	0.5 Mbps, 1 Mbps, and 4 Mbps
[103]	Remote driving using 3G	DL: avg. 121 ms video streams	N/A
[29]	Conduct remote driving prototype in a controlled lab environment. The delay is manually simulated to match LTE measured delay	100 ms for video transmission	UL: 16.02 Mbps, DL: 7.43 Mbps
[4]	14-mile drive in Scotland, UK, across the city’s outskirts, mixing urban, secondary roads, and a highway over 4G networks	Avg. video streaming 648 ms 1280 × 720 pixels and 563 ms 640 × 480 pixels video	N/A
[41]	ROS-based video streaming during remote driving, WiFi with TCP and UDP protocols	UDP video streaming 720P with 50 ms	N/A
[91]	Single-cell 5G network that covers a 3-lane highway scenario	UL 100 ms, DL 20 ms	UL: 32 Mbps, DL: 0.4 Mbps
[104]	Driving locations for multiple paths	170 ms minor impact, 700 ms significant impact.	N/A
[105]	Remote driving in a slalom course, setup with several traffic cones	3G: video 205 ms and control 217 ms; 4G: video 183 ms and control 110 ms	N/A
[21]	Parking, snake, pylon and long track	At least 300 ms	N/A
[32,106]	Direct remote steering, indirect remote driving instructions	UL: 100 ms, DL: 20 ms	UL: 32/36 Mbps, DL: 0.4 Mbps
[32,107]	Virtual digital twin remote driving	UL: 10–50 ms, DL: 10–66 ms	UL: 8–50 Mbps, DL: 5 Mbps
[32,108,109]	Remotely controlled manoeuvring, path-based driving; unexpected blockage on desired route/parking lot of vehicle, overcoming obstacle, trajectory-based driving, rural road or highway	UL: 40–80 ms	UL: 10–50 Mbps, DL: 0.5 Mbps
[32,110]	Vehicle enters a roadblock or blocking scenario	UL: 120 ms, DL: 80 ms	N/A
[24,32]	Direct remote steering, indirect remote driving instructions	300 ms	UL: 8–30 Mbps, DL: 0.3 Mbps
[32,111]	Information/support message exchange V2X, absolute speed of up to 250 km/h	UL: 5 ms, DL: 5 ms	UL: 25 Mbps, DL: 1 Mbps
[112]	Vehicle-to-everything (V2X) use cases	Maximum of 5 ms	UL: 25 Mbps, DL: 1 Mbps
[100]	Teleoperated driving with 5G	UL and DL 40 ms	UL: 3–32 Mbps, DL: 0.5 Mbps

## Data Availability

Not applicable.

## Abbreviations

The following abbreviations are used in this manuscript:

1G	First generation
3G	Third generation
3GPP	Third Generation Partnership Project
4G	Fourth generation
5G	Fifth generation
6G	Sixth generation
ADS	Automated driving system
AE	Auto-encoder
AEB	Autonomous emergency braking
AI	Artificial intelligence
ASUV	Autonomous surface and underwater vehicle
BSI	British Standards Institution
CAV	Connected and autonomous vehicle
C-ITS	Cooperative intelligent transport system
C-V2X	Cellular vehicle-to-everything
DL	Downlink
DNN	Deep neural network
DSRC	Dedicated short-range communication
DTMF	Dual-tone multi-frequency
DVI	Digital visual interface
EBs	Exabytes
ETSI	European Telecommunications Standards Institute
FOV	Field-of-view
FPS	Frames per second
GAN	Generative adversarial network
GB	Gigabytes
GEV	Generalised extreme value distribution
GPRS	General Packet Radio Service
HMI	Human–machine interface
IEEE	Institute of Electrical and Electronics Engineers
IoE	Internet of Everything
IoV	Internet of Vehicles
IPC	Inter-process communication
ITS	Intelligent transport system
ITU	International Telecommunication Union
LAN	Local area network
LCD	Liquid-crystal display
LSTM	Long short-term memory
LTE	Long-term evolution
Mbps	Megabits per second
MCW	Minimum contention window
MEC	Mobile edge computing
MHz	Megahertz
MMT	Model-mediated teleoperation
MRC	Minimal risk condition
MRM	Minimal risk manoeuvre
NN	Neural network
ODD	Operational design domain
PAS	Publicly available specification
PDF	Probability density function
QoE	Quality of experience
QoS	Quality of service
RAN	Radio access network
RF	Radio frequency
RNN	Recurrent neural network
RTMP	Real-Time Messaging Protocol
RTT	Round-trip time
SAE	Society of Automotive Engineers
SDN	Software-defined network
Seq2Seq	Sequence-to-sequence model
TDPA	Time-domain passivity analysis
TSN	Time sensitive networking
UAV	Unmanned aerial vehicle
UDP	User Datagram Protocol
UGV	Unmanned ground vehicle
UL	Uplink
V2V	Vehicle-to-vehicle
V2I	Vehicle-to-infrastructure
V2X	Vehicle-to-everything
VAE	Variational auto-encoder
Veh/km^2^	Vehicles per kilometre squared
VLAN	Virtual local area network
VLC	VideoLAN client
WiFi	Wireless fidelity
